# Interferons: Tug of War Between Bacteria and Their Host

**DOI:** 10.3389/fcimb.2021.624094

**Published:** 2021-03-10

**Authors:** Noémie Alphonse, Ruth E. Dickenson, Charlotte Odendall

**Affiliations:** ^1^Department of Infectious Diseases, School of Immunology and Microbial Sciences, King’s College London, London, United Kingdom; ^2^Immunoregulation Laboratory, Francis Crick Institute, London, United Kingdom

**Keywords:** bacterial effectors, interferons, interferon-stimulated genes, janus kinase signal transducer and activator of transcription signaling, immunity, microbial pathogenesis, host-pathogen interactions

## Abstract

Type I and III interferons (IFNs) are archetypally antiviral cytokines that are induced in response to recognition of foreign material by pattern recognition receptors (PRRs). Though their roles in anti-viral immunity are well established, recent evidence suggests that they are also crucial mediators of inflammatory processes during bacterial infections. Type I and III IFNs restrict bacterial infection *in vitro* and in some *in vivo* contexts. IFNs mainly function through the induction of hundreds of IFN-stimulated genes (ISGs). These include PRRs and regulators of antimicrobial signaling pathways. Other ISGs directly restrict bacterial invasion or multiplication within host cells. As they regulate a diverse range of anti-bacterial host responses, IFNs are an attractive virulence target for bacterial pathogens. This review will discuss the current understanding of the bacterial effectors that manipulate the different stages of the host IFN response: IFN induction, downstream signaling pathways, and target ISGs.

## Introduction

The first interferons (IFNs) were discovered in 1957 by Alick Isaacs and Jean Lindenmann when they noticed that tissues inoculated with inactivated virus produced a soluble substance that “interfered” with subsequent viral infection (Isaacs and Lindenmann, 1957). Over 20 years before Charles Janeway’s (Janeway, 1989) predictions on pattern recognition, Isaacs and Lindenmann had recognized the fundamental properties of IFN: a cytokine produced in response to detection of pathogen associated molecular patterns (PAMPs) by pattern recognition receptors (PRRs), that can protect tissues from subsequent microbial infections.

There are three families of IFN: type I, II, and III, which bind to the IFNα receptor (IFNAR), IFNγ receptor (IFNGR) and IFNλ receptor (IFNLR), respectively. Type I IFNs include IFNα, IFNβ, and the lesser-studied IFNϵ, κ, τ, δ, ζ, and ω and are expressed by almost all cells (Pestka et al., 2004; Hertzog and Williams, 2013). IFNγ is the sole type II IFN family member and is not induced in response to pattern recognition, but rather by immune cells in response to other cytokines (Pien et al., 2000; Salazar-Mather et al., 2000; Schindler et al., 2001). Type III IFNs were discovered most recently (Sheppard et al., 2002; Kotenko et al., 2003) and include four members in humans: IFNλ1-4. IFNAR and IFNGR are expressed on all nucleated cells, however the expression of IFNLR is restricted to epithelial cells (ECs) and some immune cells, including neutrophils (Blazek et al., 2015; Broggi et al., 2017). As such, type I and II IFNs have systemic functions (De Weerd and Nguyen, 2012; Chen et al., 2017; Lazear et al., 2019) while type III IFNs are crucial for host defense at barrier sites including the gut and lung (Durbin et al., 2013; Broggi et al., 2017; Lazear et al., 2019; Broggi et al., 2020). IFNs signal in an autocrine and paracrine manner through their respective receptors to activate janus kinase/signal transducer and activator of transcription (JAK/STAT) signaling cascades, resulting in the expression of hundreds of genes, known collectively as interferon-stimulated genes (ISGs). Although the roles of IFNs and ISGs have been extensively studied in the context of viral infection, there is a dearth of knowledge of their role during bacterial infections. Type I and III IFNs are strongly induced upon recognition of bacterial ligands, and play diverse and context-dependent roles during infection (Kagan et al., 2008; Pandey et al., 2009).

To promote their survival within the host, bacteria have evolved virulence factors such as secretion systems; apparatuses that translocate effector proteins across host membranes (Green and Mecsas, 2016). These effectors enable the pathogen to evade and perturb the host response or even use it to their advantage. This review will examine the interplay between bacterial effectors and the IFN response. As modulation of IFNγ has been previously discussed (Kak et al., 2018) we have focussed our discussion on the lesser understood type I and III IFNs. We will first summarize the current understanding of the complex functions of type I and III IFNs in the defense against pathogenic bacteria.

## The Capricious Roles of Types I and III Interferons During Bacterial Infections

Type I and III IFNs have anti-bacterial properties in most *in vitro* tissue culture models. For example, treatment of polarized ECs by type I and/or III IFNs protects epithelial barriers from damage caused by enteropathogenic *Escherichia coli* (EPEC) (Long et al., 2014), *Salmonella enterica* serovar Typhimurium, and *Shigella flexneri* (Odendall et al., 2017). In addition, type I IFN restricts the replication of several intracellular bacteria, including *S*. Typhimurium and *S. flexneri* (Bukholm et al., 1984; Niesel et al., 1986; Duménil et al., 1998; Helbig et al., 2019), *Chlamydia trachomatis* (Snyder et al., 2017), *Mycobacterium tuberculosis* (Ranjbar et al., 2015), *Legionella pneumophila* (Lippmann et al., 2011), *Listeria monocytogenes* (Zwaferink et al., 2008; Radoshevich et al., 2015), *Francisella novicida* (Henry et al., 2007; Henry et al., 2010), and *Rickettsia parkeri* (Burke et al., 2020). The inhibition of bacterial replication is often the result of the action of ISGs. For example, viperin is a highly evolutionarily conserved ISG that restricts *S. flexneri* infection in HeLa cells (Helbig et al., 2019).

The roles of type I and III IFNs are more complex to delineate *in vivo* and can vary depending on the pathogen and biological context. Type I IFNs can restrict infections by pathogens such as *Bacillus anthracis* (Walberg et al., 2008), *L. pneumophila* (Lippmann et al., 2011; Naujoks et al., 2016), *Helicobacter pylori* (Watanabe et al., 2010), *Streptococcus pyogenes* (Castiglia et al., 2016), group B *Streptococcus*, *E. coli* (Mancuso et al., 2007), and *Streptococcus pneumoniae* (LeMessurier et al., 2013). In particular, IFNs prevented bacterial migration across endothelial and epithelial barriers (LeMessurier et al., 2013). Whether this protection extends to type III IFNs is unclear, but type III IFNs were demonstrated to be protective in murine models of colitis (Rauch et al., 2015; Broggi et al., 2017). In contrast, in other infection models, type I IFNs can be detrimental to hosts infected with bacterial pathogens. When compared to wild-type (WT) mice, *Ifnar^−/−^* animals were more resistant to systemic infection with *L. monocytogenes* (Auerbuch et al., 2004; Brzoza-Lewis et al., 2012), and *S*. Typhimurium (Robinson et al., 2012; Perkins et al., 2015; Wilson et al., 2019; Zhang et al., 2020). Similarly, IFNβ treatment exacerbated infection with *M. tuberculosis* (Manca et al., 2001; Manca et al., 2005) and *B. anthracis* (Gold et al., 2007). The detrimental nature of type I IFN responses during *M. tuberculosis* infection is reflected in human disease. The blood transcriptome of patients with active disease has demonstrated a correlation between the abundance of Type I IFN-inducible transcripts and disease pathogenesis (Berry et al., 2010; Maertzdorf et al., 2011; Ottenhoff et al., 2012; Cliff et al., 2013). In addition, individuals with an inherited defect in ISG15 have an increased susceptibility to mycobacterial, but not viral, disease (Bogunovic et al., 2012).

Finally, type I and III IFNs induced by viral infection were shown to exacerbate subsequent respiratory superinfections with *S. pneumoniae* or *Staphylococcus aureus*. Indeed, *Ifnar^−/−^* and *Ifnlr^−/−^* mice showed improved bacterial control in virus-bacteria superinfection models (Shahangian et al., 2009; Broggi et al., 2020; Major et al., 2020). Similarly, administration of recombinant type I or III IFN resulted in increased bacterial burdens following viral infection or activation of antiviral pathways with viral ligands. Recent work has shown that uncontrolled type III IFN responses, such as those observed during SARS-CoV-2 infection, led to damage of epithelial barriers and increased susceptibility to bacterial superinfection, a known complication of COVID19 and Influenza (Broggi et al., 2020; Langford et al., 2020; Major et al., 2020).

## Targeting of Types I and III Interferon Expression by Bacterial Effectors

In order to control their host, pathogenic bacteria secrete effectors that manipulate different stages of the IFN response, from its production to signaling and even ISG functions.

Host recognition of bacterial PAMPs occurs *via* PRR ligation, leading to the assembly of signaling complexes and the activation of intracellular adaptor proteins [reviewed in (Odendall and Kagan, 2017)]. Cytosolic RNA sensors, retinoic acid-inducible gene-I (RIG-I)-like receptors (RLRs), signal *via* the adaptor mitochondria-antiviral signaling protein (MAVS) (Kawai et al., 2005; Meylan et al., 2005; Seth et al., 2005; Xu et al., 2005; Dixit et al., 2010), while the DNA sensor cyclic GMP–AMP synthase (cGAS) signals *via* stimulator of interferon genes (STING), localized on the endoplasmic reticulum (ER) (Gao et al., 2012; Wu et al., 2017). Toll-like receptors (TLRs) present on the cell surface and endosomes, signal *via* the adaptors MyD88 and/or TRIF (Kawasaki and Kawai, 2014). Although only endosomal TLRs efficiently induce type I IFNs, all TLRs were shown to strongly induce type III IFNs in response to bacterial ligands (Odendall et al., 2017). These signaling pathways culminate in the activation and nuclear translocation of transcription factors, including interferon regulatory factors (IRFs) that control IFN expression (Takeuchi and Akira, 2010; Odendall and Kagan, 2017; Odendall and Kagan, 2019). IFN expression also requires the NFκB and MAP-kinase activated transcription factor AP-1 (Odendall and Kagan, 2017).

**Figure 1:**
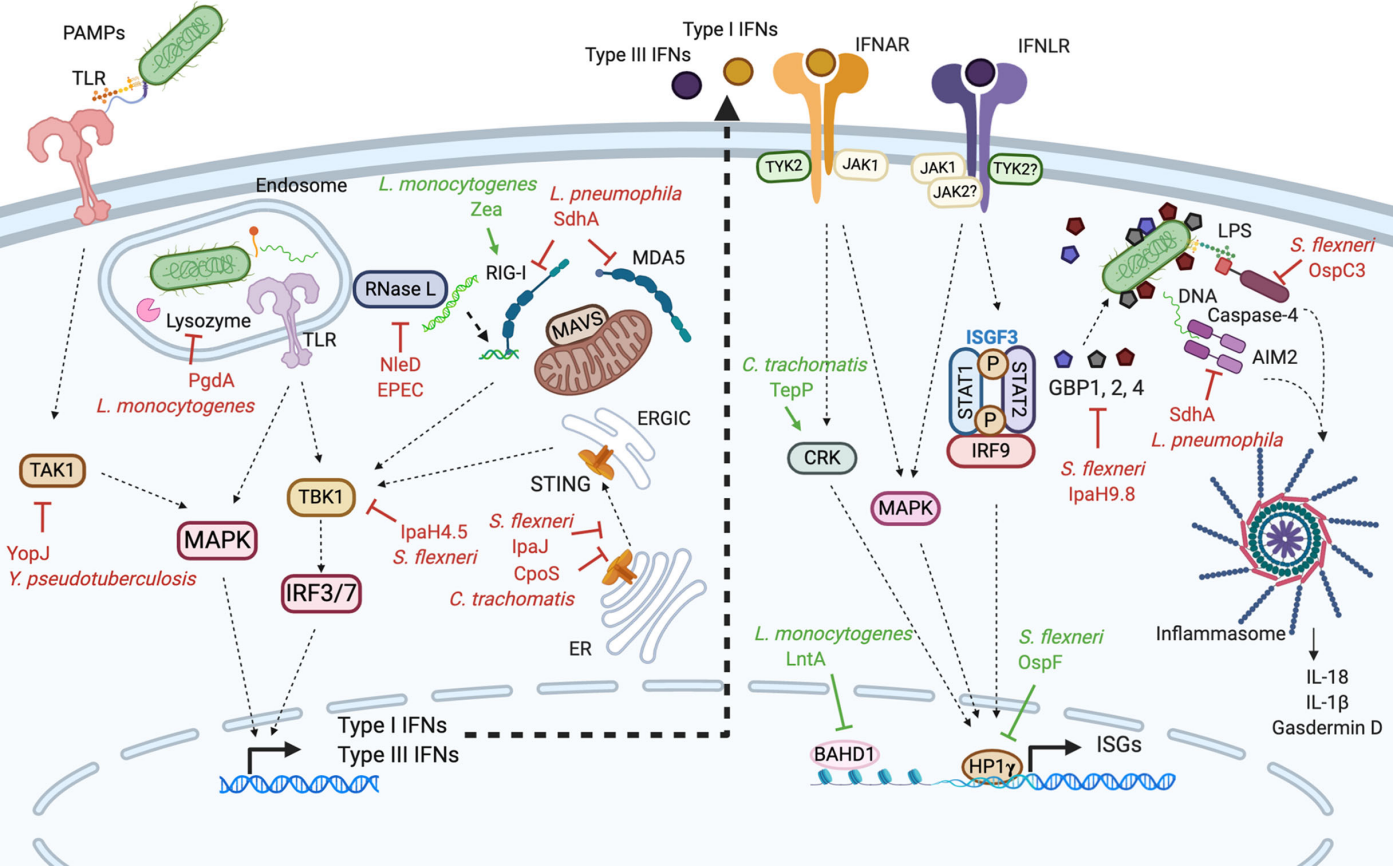
Modulation of types I and III interferon responses by bacterial effectors. In many different cell types, the recognition of bacterial PAMPs by PRRs leads to the production of type I and III IFNs (left side). Type I and III IFNs bind their respective receptors initiating signaling pathways which trigger the expression of ISGs (right side). Bacteria have evolved effectors that either inhibit (depicted in red) or stimulate (depicted in green) type I and III IFN responses. *Yersinia pseudotuberculosis* YopJ effector interferes with the MAP kinase activator TAK1. When internalized in endosomes, *Listeria monocytogenes* secretes PgdA which modifies its peptidoglycan, hiding it from the host lysozyme. *Listeria monocytogenes* also secretes Zea which binds RIG-I and potentiates the production of IFNs. The *Legionella pneumophila* protein SdhA blocks RIG-I and MDA5 cytosolic PRRs preventing them to interact with MAVS. The *Shigella flexneri* protease IpaJ interferes with STING translocation to the ERGIC while IpaH4.5 inhibits TBK1 kinase. Similarly, *Chlamydia trachomatis* CpoS effector affects STING migration. EPEC, through the secretion of NleD inhibits RNase L which counteracts the protective effects of type I IFN on epithelial barriers. Additionally, some bacteria perturb type I and III IFN signaling. *L. monocytogenes* LntA and *S. flexneri* OspF effectors enter the cell nucleus and potentiate ISGs transcription. LntA sequesters BAHD1, a negative regulator of ISGs transcription therefore stimulating ISGs expression. OspF interacts with HP1γ preventing its phosphorylation, which promotes ISGs expression. *C. trachomatis* TepP effector inhibits CRK, a component and regulators of type I and III IFN signaling, stimulating ISGs transcription. Finally, *S. flexneri* IpaH9.8 and OspC3 as well as *L. pneumophila* SdhA effectors inhibit inflammasome components; GBP1, caspase-4, and AIM2 respectively. PRRs, pattern recognition receptors; PAMPs, pathogen-associated molecular patterns; TLR, Toll like receptor; STING, stimulator of interferon genes; ER, endoplasmic reticulum; ERGIC, ER-Golgi intermediate compartments; IRF, interferon regulatory factor; RIG-I, retinoic acid-inducible gene-I; MDA5, melanoma differentiation-associated protein 5; MAVS, mitochondria-antiviral signaling protein; TAK1, TGFbeta activated kinase 1; TYK2, tyrosine kinase 2; JAK1, Janus kinase 1; STAT, signal transducer and activator of transcription; ISGF3, interferon-stimulated gene factor 3; MAPK, mitogen-activated protein kinase; GBP, guanylate-binding protein; AIM2, absent in melanoma-2; LPS, lipopolysaccharide; ISG, interferon stimulated gene; IFNAR, IFNα receptor; IFNLR,IFNλ receptor. **Figure 1** was created with Biorender.com.

As IFNs can have both beneficial and detrimental effects on bacterial pathogens, some species have evolved effectors that promote or inhibit their production (summarized in **Figure 1** and **Table 1**). For example, *L. monocytogenes* secretes *Zea*, a ribonucleoprotein, that binds RIG-I and potentiates type I IFN responses in ECs (Pagliuso et al., 2019). In contrast, many bacterial effectors block the induction of IFN. The effects of some of these effectors on IFN induction may be considered as indirect as they either interfere with PAMP detection or block general innate sensing pathways. *L*. *monocytogenes* secretes an effector, PgdA, that deacetylates peptidoglycan, conferring a resistance to host lysozyme. This prevents the release of PAMPs and dampens the induction of cytokines and type I IFN (Boneca et al., 2007). Similarly, the *L. pneumophila* effector SdhA inhibits RLR activation in mouse Bone marrow-derived macrophage (BMDM) by maintaining the integrity of the *Legionella*-containing vacuole (Monroe et al., 2009). The cGAS/STING pathway has since been shown to mediate IFN expression following *Legionella* infection (Ruiz-Moreno et al., 2018). Although this has not been formally tested, it is likely that SdhA also protects from detection by cGAS. *Yersinia pseudotuberculosis* YopJ targets MAP-kinase signaling in dendritic cells and macrophages, disrupting MyD88- and TRIF-dependent signaling downstream of TLR4, which prevents type I IFN and pro-inflammatory cytokine expression (Paquette et al., 2012; Rosadini et al., 2015). EPEC is able to counteract the protective effects of type I IFN on epithelial barriers; its effector NleD inhibits RNase L, an endoribonuclease that enhances RLR-mediated production of IFNβ (Long et al., 2014).

**Table 1:** Summary of effectors that target IFN pathways.

Bacteria	Effector	Secretion system	Biochemical activity	Function	References
***Bacillus anthracis***	UN	T4SS-like	UN	Inhibits the assembly of ISGF3	(Gold et al., 2004)
***Chlamydia pneumoniae***	UN	T3SS	Protease	Degrades TRAF3	(Wolf and Fields, 2013)
***Chlamydia trachomatis***	CpoS	T3SS	UN	Counteracts STING-mediated IFN responses and perturbs ISRE and ISG expression	(Sixt et al., 2017)
***Chlamydia trachomatis***	TepP	T3SS	Scaffold	Binds Crk-I and Crk-II, initiating and amplifying signaling cascades (*IFIT1*, *IFIT2*)	(Chen et al., 2014)
***Enteropathogenic Escherichia coli***	NleD	T3SS	Zinc metallo-protease	Targets the endoribonuclease RNase L, a key mediator of IFNβ induction and action, compromising the barrier integrity	(Long et al., 2014)
***Legionella pneumophila***	SdhA	T4SS	UN	Helps maintain vacuolar integrity	(Monroe et al., 2009) (Creasey and Isberg, 2012)
		T4SS	UN	Negatively regulates AIM2 inflammasome activation	(Ge et al., 2012)
***Listeria monocytogenes***	LntA	Sec SS	Nucleo-modulin	Interacts with BAHD1, inducing ISG expression	(Lebreton et al., 2011)
	PgdA	?	Peptido-glycan N-deacetylase	Deacetylates bacterial peptidoglycan, conferring a resistance to host lysozyme which ultimately prevents the release of PAMPs	(Boneca et al., 2007)
	Zea	Sec SS	Ribonucleo-protein	Triggers a RIG-I dependent type I IFN response	(Pagliuso et al., 2019)
***Salmonella* Typhimurium**	UN	T3SS	UN	Represses the TRIF-dependent type I IFN response	(Owen et al., 2016)
***Shigella flexneri***	IpaJ	T3SS	Cysteine protease	Blocks STING translocation from the ER to the ERGIC compartment	(Dobbs et al., 2015)
	IpaH4.5	T3SS	E3 ubiquitin ligase	Promotes proteasome-dependent degradation of TBK1	(Zheng et al., 2016)
	OspC3	T3SS	UN	Binds and inhibits Caspase 4	(Kobayashi et al., 2013)
	OspF	T3SS	Phospho-threonine lyase	Inhibits HP1γ; phosphorylation, repressing the expression of some IFN-regulated genes: *Ifi44*, *Ifit3*, and *Oas1b*	(Lavigne et al., 2009; Harouz et al., 2014)
	IpaH9.8	T3SS	E3 ubiquitin ligase	Targets GBP1, GBP2 and GBP4 for proteasomal degradation	(Li et al., 2017; Wandel et al., 2017)
***Yersinia pseudo-tuberculosis***	YopJ	T3SS	De- ubiquitinase/Acetyl transferase	Targets TAK1 to inhibit IFN production	(Rosadini et al., 2015) (Paquette et al., 2012)

UN, unknown.

Other effectors affect the localization of adaptor proteins that are crucial to PRR signaling cascades. For example, STING translocates from the ER to the ER-Golgi intermediate compartments (ERGIC) to mediate cGAS signaling (Ishikawa et al., 2009; Burdette and Vance, 2013). IpaJ is a *S. flexneri* protease that targets the small guanosine triphosphatases (GTPases) required for this migration event. This blocks STING translocation, abolishing type I IFN and ISG expression (Dobbs et al., 2015). Similarly CpoS from *C. trachomatis* affects STING migration and blocks type I and III IFN and ISG induction (Sixt et al., 2017).

The kinase TBK1 lies downstream of many PRRs and phosphorylates IRF3 and IRF7, leading to the expression of type I and III IFNs (Clark et al., 2011; Tanaka and Chen, 2012; Schneider et al., 2014; Liu et al., 2015; Bakshi et al., 2017). It can also activate IRF1, which may specifically drive type III IFN induction (Odendall et al., 2014). The *S. flexneri* effector IpaH4.5 is an E3 ubiquitin ligase that targets TBK1 for proteasomal degradation, leading to the suppression of IFN production (Zheng et al., 2016). Interestingly, mice infected with Δ*ipaH4.5* mutants had lower bacterial burdens than those infected with WT *S. flexneri*.

Finally, some bacteria modulate IFN production *via* yet-unknown mechanisms. *Salmonella* represses TRIF-dependent type I IFN induction in macrophages (Owen et al., 2016). Likewise, *Chlamydia pneumoniae* infection of ECs does not lead to IFNβ production and very efficiently blocks IRF3 phosphorylation. Although the effector responsible was not identified, *Chlamydia* was shown to induce the degradation of TRAF3, a crucial TBK1 activator (Wolf and Fields, 2013). Conversely, some pathogens such as *Legionella* (Ruiz-Moreno et al., 2018) or *Burkholderia pseudomallei* (Ku et al., 2020), were shown to induce IFNβ by activating the cGAS-STING pathway in a secretion system-dependent manner.

Although the pathways that drive type I and III IFN expression overlap significantly, the majority of this research has focused on type I IFNs. It will be interesting to investigate whether bacterial effectors can specifically target the production of one family or the other.

## Effector-Driven Manipulation of Signaling Downstream of Interferon Receptors

JAK/STAT signaling activated by type I or III IFN receptor binding leads to the formation of a STAT1/STAT2/IRF9 complex called the interferon-stimulated gene factor 3 (ISGF3). This complex translocates to the nucleus and binds interferon stimulated response elements (ISREs) to transcribe ISGs. Instead of targeting IFN production, some bacterial effectors target signaling cascades downstream of IFN receptors to alter the expression of ISGs. For example, *B. anthracis* inhibits IFNβ-mediated STAT1 phosphorylation and the formation of ISGF3, but the bacterial effectors remain to be identified (Gold et al., 2004).

Bacteria can also affect transcription of ISGs by altering epigenetic regulators of transcription. Heterochromatin protein 1 (HP1) family members are epigenetic regulators that bind methylated histone H3 to influence the expression of a wide variety of genes (Saint-André et al., 2011; Ameyar-Zazoua et al., 2012; Smallwood et al., 2012). In a guinea pig model of *Shigella* enterocolitis, *S. flexneri* inhibited HP1γ phosphorylation *via* OspF. Next-generation sequencing of mouse embryonic fibroblast-derived cell lines revealed that HP1γ represses the expression of some IFN-regulated genes, namely *Ifi44*, *Ifit3*, and *Oas1b* (Harouz et al., 2014), confirming previous observations (Lavigne et al., 2009). This suggests that *S. flexneri* effectors are capable of modifying epigenetic regulators of subsets of ISGs to alter the outcome of IFN signaling. Likewise, the *C. trachomatis* effector TepP, was shown to bind the adaptor protein CRK and affect expression of ISGs in human cell lines (Chen et al., 2014).

Some bacterial effectors enter the nucleus to alter chromatin remodeling in their mammalian hosts (Bhavsar et al., 2007; Lebreton et al., 2011). Infection of cells with an *L. monocytogenes* strain constitutively expressing the effector LntA resulted in enrichment of expression of a number of genes, 83% of which were ISGs. Interestingly, when *lntA* was deleted, there was a reduction in bacterial burden in the spleens and livers of mice. LntA was shown to interact with BAHD1; a heterochromatin protein that acts as a transcriptional repressor (Bierne et al., 2009). Chromatin immunoprecipitation analysis showed that association of BAHD1 with key ISGs was reduced in cells infected with *L. monocytogenes* constitutively expressing LntA. Therefore, *Listeria* induces the expression of ISGs by inhibiting the negative regulator BAHD1 (Lebreton et al., 2011).

## Bacterial Effectors and Type I and III Interferon-Stimulated Genes

All three families of IFNs induce the expression of ISGs. As discussed above, the set of 300–600 genes transcribed by type I and III IFNs contain ISRE sequences. In contrast, IFNγ induces ISGs containing a gamma interferon activation site (GAS) element. ISGs can contain both ISRE and GAS sequences and therefore be induced by all three families. As they are induced in concert, the individual roles of many ISGs are not well understood. Although more is known about the ISGs targeted by viral virulence factors, there are few examples of bacterial factors that can alter their integrity or function.

Among ISGs are several components of inflammasomes. These are large multi-protein complexes that are assembled upon recognition of PAMPs or changes in host homeostasis. Inflammasome activation leads to pyroptosis, a highly inflammatory form of cell death. Inflammasomes are key to host defense against intracellular bacteria. As such, some bacteria have evolved strategies to evade inflammasome activation. For example, absent in melanoma-2 (AIM2) is an IFN-inducible inflammasome receptor that detects the presence of bacterial DNA in the cytosol (Man et al., 2015; Meunier et al., 2015). The *L. pneumophila* effector SdhA, in addition to suppressing type I IFN responses (Monroe et al., 2009; Creasey and Isberg, 2012), negatively regulates AIM2 inflammasome activation in human macrophages (Ge et al., 2012).

Another inflammasome ‘receptor’ induced by IFNs is murine caspase 11, which detects intracellular LPS and induces the non-canonical inflammasome (Ding and Shao, 2017). The human orthologs of caspase 11 are caspases 4 and 5, but although caspase 4 was shown to be transcriptionally induced by type I IFN, whether this occurs at the protein level is still poorly understood (Knodler et al., 2014; Casson et al., 2015; Schmid-Burgk et al., 2015). However, it is clear that the caspase 4 inflammasome is “primed” by IFN *via* the action of guanylate-binding proteins (GBPs) (Pilla et al., 2014; Feng and Man, 2020; Santos et al., 2020). GBPs are among the best-characterized antibacterial ISGs. This family of GTPases has an ever-growing list of functions, many of which contribute to defenses against intracellular pathogens. There are seven *GBP* genes in humans, whose expression is most robustly induced by IFNγ, but can also be induced by treatment with type I IFNs (Cheng et al., 1985; Kim et al., 2011; Tretina et al., 2019). Some GBPs can also be produced in response to treatment with type III IFNs (Alase et al., 2015; Tretina et al., 2019). GBPs are recruited to intracellular gram-negative bacteria (*S*. Typhimurium, *S. flexneri*, *Burkholderia thailandensis*, *Brucella abortus*, *F. novicida*, *C. trachomatis*, and *L. pneumophila*) and liberate LPS into the cytosol (Kim et al., 2011; Kim et al., 2012; Shenoy et al., 2012; Haldar et al., 2014; Meunier et al., 2014; Man et al., 2015; Meunier and Broz, 2015; Pilla et al., 2014; Shi et al., 2014; Man et al., 2016; Feeley et al., 2017; Finethy et al., 2017; Lindenberg et al., 2017; Cerqueira et al., 2018; Fisch et al., 2019; Bass and Shin, 2020; Santos et al., 2020). Recent work on *S. flexneri* and *S*. Typhimurium has shown that GBP1 directly binds bacterial LPS through electrostatic interactions and assembles a signaling platform containing other GBPs. This platform recruits and activates human caspase 4, leading to assembly of the non-canonical inflammasome (Kutsch et al., 2020; Santos et al., 2020; Wandel et al., 2020). In addition, GBPs inhibit intracellular motility and cell-to-cell spread of *B. thailandensis* and *S. flexneri* (Ostler et al., 2014; Piro et al., 2017; Wandel et al., 2017).

*S. flexneri* has evolved at least two effector proteins to bypass these important host defense mechanisms; firstly, OspC3, which binds and inhibits caspase 4. *ospC3* mutants have a growth defect in IFNγ-treated cells and induce rapid cell death. These mutants were also attenuated in WT mice, but not in mice lacking GBP1, GBP2, or caspase 11 (Kobayashi et al., 2013; Wandel et al., 2020). Whether they are more sensitive to type I or III IFNs is unclear. Secondly, the E3 ubiquitin ligase IpaH9.8 targets hGBP1, hGBP2, and hGBP4 for proteasomal degradation (Li et al., 2017; Wandel et al., 2017). *Shigella* strains lacking *ipaH9.8* displayed an increased recruitment of GBP1, 2, 3, and 4, as well as caspase 4, compared to WT (Wandel et al., 2017; Wandel et al., 2020). In addition, cell-to-cell spread of *ipaH9.8* mutant bacteria was lowered (Wandel et al., 2017). These data highlight the importance of GBPs to entrap intracellular bacteria and act as platforms for the activation of innate signaling. It is therefore highly probable that other intracellular bacteria have evolved mechanisms to counteract GBPs.

## Concluding Remarks

IFNs are crucial mediators of inflammation, playing complex, yet key roles in both systemic and localized bacterial infections. In order to survive and cause disease, bacteria secrete effectors that interfere with type I and III IFN production and signaling, as well as ISG expression and function. As there is still much to learn about the role of IFN and ISGs, perhaps understanding the mechanisms that bacterial pathogens have evolved to evade or enhance these responses will lead to insights into their function in host defense. These discussions certainly mandate further studies into the interactions between IFNs and bacteria.

## Author Contributions

NA and RD contributed equally. All authors contributed to the article and approved the submitted version.

## Funding

NA is supported by a studentship from the King’s College London/Francis Crick Institute partnership, which receives its core funding from the Cancer Research UK (FC001206), the UK Medical Research Council (FC001206), and the Wellcome Trust (FC001206). RD is supported by a studentship from the UK Medical Research Council (MR/N013700/1). CO is supported by a Sir Henry Dale Fellowship from the Royal Society and the Wellcome Trust (206200/Z/17/Z).

## Conflict of Interest

The authors declare that the research was conducted in the absence of any commercial or financial relationships that could be construed as a potential conflict of interest.
